# Practice Gap in Atrial Fibrillation Oral Anticoagulation Prescribing at Emergency Department Home Discharge

**DOI:** 10.5811/westjem.2020.3.45135

**Published:** 2020-06-29

**Authors:** Bory Kea, Bethany T. Waites, Amber Lin, Merritt Raitt, David R. Vinson, Niroj Ari, Luke Welle, Andrew Sill, Dana Button, Benjamin C. Sun

**Affiliations:** * Oregon Health & Science University, Center for Policy and Research—Emergency Medicine, Department of Emergency Medicine, Portland, Oregon; † Kaiser Permanente, Department of Obstetrics and Gynecology, San Francisco, California; ‡ Oregon Health & Science University, Knight Cardiovascular Institute, VA Portland Health Care System, Portland, Oregon; § The Permanente Medical Group and Kaiser Permanente Division of Research, Oakland, California; ¶ Portland State University, School of Public Health, Portland, Oregon; || Oregon Health & Science University, School of Medicine, Department of Emergency Medicine, Portland, Oregon; # University of Pennsylvania, Department of Emergency Medicine, Leonard Davis Institute of Health Economics, Philadelphia, Pennsylvania

## Abstract

**Introduction:**

Current U.S. cardiology guidelines recommend oral anticoagulation (OAC) to reduce stroke risk in selected patients with atrial fibrillation (AF), but no formal AF OAC recommendations exist to guide emergency medicine clinicians in the acute care setting. We sought to characterize emergency department (ED) OAC prescribing practices after an ED AF diagnosis.

**Methods:**

This retrospective study included index visits for OAC-naive patients ≥18 years old who were discharged home from the ED at an urban, academic, tertiary hospital with a primary diagnosis of AF from 2012–2014. Five hypothesis-blinded, chart reviewers abstracted data from patient problem lists and medical history in the electronic health record to assess stroke (CHA_2_DS_2_-VASc) and bleeding risk (HAS-BLED). The primary outcome was the provision of an OAC prescription at discharge in OAC-naive patients with high stroke risk. Descriptive statistics and multivariable logistic regression assessed associations between OAC prescription and patient characteristics.

**Results:**

We included 138 patient visits in our analysis, of whom 39.9% (n = 55) were low stroke risk (CHA_2_DS_2_-VASc = 0 in males and 1 in females), 15.9% (n = 22) were intermediate risk (CHA_2_DS_2_-VASc = 1 in males), and 44.2% (n = 61) were high risk (CHA_2_DS_2_-VASc ≥ 2). Of patients with high stroke risk and low-to-intermediate bleeding risk (n = 57), 80.7% were not prescribed an OAC at discharge. Cardiology consultation and female gender, but not stroke risk (CHA_2_DS_2_-VASc score), were predictors of an ED provider prescribing an OAC to an OAC-naive AF patient at ED discharge.

**Conclusion:**

The majority of OAC-eligible patients were discharged home without an OAC prescription. In OAC-naive patients discharged home from the ED, cardiology consultation and female gender were associated with OAC prescription. Our findings suggest that access to expert opinion may improve provider comfort with OAC prescribing and highlight the need for improved guidelines specific to ED-management of AF.

## INTRODUCTION

Atrial fibrillation (AF) is the most common arrhythmia presenting to emergency departments (EDs) and accounts for more than 500,000 annual ED visits; up to one quarter of all new AF diagnoses are made in the ED.^1^–^2^ The related costs for these patients total more than $26 billion annually.^3^ Importantly, AF has significant associated morbidity and mortality,^4^ with a fivefold increase in an individual’s lifetime risk of stroke when compared to a non-AF reference population.^5^–^7^ Compared to estimates from 2010, the prevalence and incidence of AF are both expected to double by the year 2030, when over 12 million Americans will be affected.^8^

Although studies show that oral anticoagulation (OAC) therapy with traditional agents such as warfarin or non-vitamin K oral anticoagulants (NOACs) can reduce stroke risk by 64% in non-valvular AF, providers hesitate to prescribe OACs for reasons that include increased bleeding risk.^7^,^9^–^11^ Professional guidelines recommend the use of CHA_2_DS_2_-VASc, a validated scoring system that stratifies patients’ annual stroke risk based on age, gender, and comorbid conditions, and HAS-BLED, a complementary scoring system that predicts the likelihood of a major bleeding event in anticoagulated patients, to determine appropriate OAC recommendations.^12^–^15^

Multiple studies show a net positive clinical benefit for OAC prophylaxis in AF patients with at least one additional risk factor for stroke.^7^,^16^–^22^ With rising pressure to decrease unnecessary hospitalizations, up to 89% of patients with new-onset AF may be discharged from the ED.^17^ ED providers may defer OAC initiation for a patient with new AF to an outpatient provider, but more than half of AF patients discharged from the ED fail to achieve outpatient follow-up within 90 days of hospital discharge.^17^,^19^ Thus, ED management at discharge may determine the trajectory of care and impact clinical outcomes.

The objective of this study was to describe baseline ED OAC prescribing rates for eligible OAC-naive AF patients, characterize predictors of OAC prescribing, and identify variation from established guidelines and risk-stratification tools. This information will inform future interventions to improve prescribing in the ED and, ultimately, clinical outcomes for AF patients.

## METHODS

### Study Design and Setting

This retrospective study took place at an academic, tertiary care hospital ED with an affiliated emergency medicine (EM) residency program staffed by 43 board-certified faculty and EM residency-trained fellows with an annual ED volume of 50,000 adult patients. The study was approved by the Oregon Health & Science University Institutional Review Board.

**Population Health Research Capsule**

What do we already know about this issue?

*Up to one quarter of all new atrial fibrillation (AF) diagnoses are made in the emergency department (ED), and AF accounts for more than 500,000 annual ED visits*.

What was the research question?

What factors influence emergency physician oral anticoagulant (OAC) prescription rates for patients with a primary diagnosis of AF at home discharge?

What was the major finding of the study?

*The majority of patients were not prescribed an OAC. Cardiology consultation and female gender were associated with OAC prescription*.

How does this improve population health?

*ED-specific guidelines and access to expert opinion may improve time to OAC prescription for OAC-naive AF and reduce the associated morbidity and mortality*.

### Selection of Participants

A query of the electronic health record (EHR) identified patients ≥ 18 years old who were evaluated in the ED between January 1, 2012–December 31, 2014, and given a primary diagnosis of AF (International Classification of Disease-9 code 427.31) and discharged home from the ED. We excluded patients who were taking warfarin or a NOAC at the time of presentation. Patients taking aspirin at the time of presentation were considered OAC-naive, as aspirin is not recommended for those at high risk for stroke.^15^ Only the first eligible visit during the study period was included.

### Data Collection and Processing

We collected patient data for all qualifying patient encounters using the abstraction criteria described by Kaji et al.^23^ Four chart abstractors blinded to the study hypotheses performed the chart review. The principal investigator trained each abstractor and provided them with standardized data collection procedures and definitions. A random sample of 10 encounters was selected for re-abstraction to determine inter-rater reliability. We assessed Fleiss’ kappa and intraclass correlation statistics.

Study data were collected and managed using Research Electronic Data Capture (REDCap) electronic data capture tools. REDCap is a secure, web-based application designed to support data capture for research studies that is endorsed for clinical research purposes by institutions including Oregon Health & Science University.^24^ Abstracted data included patient demographics, risk factors for stroke/bleeding,^12^,^15^,^25^ other comorbidities documented within one year of the ED encounter, substance use (alcohol, tobacco, illicit drug use), current medication use (OACs, antiplatelets, diuretics, heart rate-controlling medications), and disabilities or trouble with activities of daily living documented within the last year. Abstracted data related to management in the ED included chief complaint at time of presentation, arrhythmia management attempted in the ED, provision of OAC/antiplatelet prescription or adjustment to antiplatelet, specialty consultations obtained by the ED provider and recommendations for anticoagulation, reason from provider for management decisions, patient disposition, and follow-up international normalized ratio (INR) (if applicable). (See Appendix for further details of data captured.)

### Outcome Measure

The primary outcome was the provision of an OAC prescription at home discharge in OAC-naive patients with AF and a high stroke risk (CHA_2_DS_2_-VASc ≥ 2). OACs included warfarin and NOACs (factor Xa and thrombin inhibitors). Based on investigator consensus, we simplified the indications for stroke prophylaxis to those who would be most acceptable by ED providers: AF patients with high stroke risk by CHA_2_DS_2_-VASc^12^ (scores ≥ 2) and low bleeding risk by HAS-BLED^25^ (scores 0–2), where AF patients would derive the greatest benefit and the least amount of harm from an OAC prescription. Although a high HAS-BLED score does not preclude the use of OACs, we chose to exclude them from the OAC indicated cohort to simplify the analysis to the most obvious cohort needing OACs with minimal concerns of adverse events for the risk-averse emergency provider.

### Variables

We identified predictor variables to compare patients prescribed an OAC upon discharge from the ED to those who were not prescribed an OAC. Variables were selected based on the reviewed literature and factors thought to impact clinical decision making, and included the following: calculated CHA_2_DS_2_-VASc and HAS-BLED scores stratifed into low, intermediate and high risk; health insurance; gender; disabilities; cardiology consultation; return to normal sinus rhythm at disposition; whether cardioversion was attempted in the ED; and first method of rate or rhythm control attempted. All were identified through review of the ED provider and consultant notes as well as encounter registration data.

We also compared patients who received a cardiology consult in the ED to those who did not in order to identify predictors of specialty consultation. Selected variables included the following: duration of symptoms; health insurance; and comorbidities used to calculate the CHA_2_DS_2_-VASc score (congestive heart failure, hypertension, age, diabetes, gender, stroke/transient ischemic attack [TIA], vascular disease). For the patients who received a cardiology consultation, we determined whether cardiology’s recommendation regarding OAC provision agreed with whether the emergency physician prescribed an OAC and identified any documented reason for discrepancy.

We documented whether or not the emergency physician cited use of a clinical guideline (such as CHA_2_DS_2_-VASc or HAS-BLED) in his or her clinical decision-making process. Similarly, we identified emergency physicians’ reasons for lack of OAC prescription in OAC-eligible patients. Lastly, we evaluated OAC and NOAC prescribing trends to investigate whether physician familiarity with newer drugs influenced prescribing of an anticoagulant.

### Statistical Analysis

Descriptive statistics were used to summarize age, race, ethnicity, insurance, the reason for evaluation, medications at the time of the encounter, CHADS_2_ score, CHA_2_DS_2_-VASc score, HAS-BLED score, and follow-up instructions. We used multivariable logistic regression to identify factors associated with provision of OAC prescription at ED discharge and also to identify factors associated with cardiology consultation. Model diagnostics were visually inspected for outliers and leverage values. All tests were two-sided with a significance level of 0.05. The analysis was conducted with SAS 9.4 (Cary, NC, USA).

## RESULTS

### Characteristics of Study Subjects

**Figure 1:**
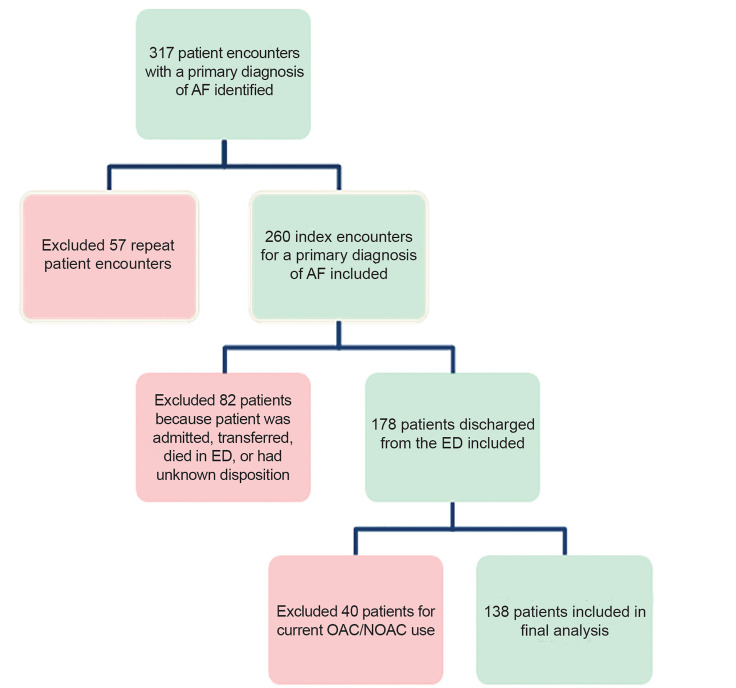
Cohort selection of patients with atrial fibrillation. *AF*, atrial fibrillation; *ED*, emergency department; *OAC*, oral anticoagulant; *NOAC*, non-vitamin K oral anticoagulants.

During the study period, 317 patients were identified, with 138 ultimately meeting inclusion criteria (Figure 1). Their baseline characteristics are reported in Table 1.

Their mean age was 59 years, 39.1% were female, and 39.9% had no history of AF. Overall, 39.9% (n = 55) were low risk for stroke (CHA_2_DS_2_-VASc = 0 in males and 1 in females), 15.9% (n = 22) were intermediate risk (CHA_2_DS_2_-VASc = 1 in males), and 44.2% (n = 61) were high risk (CHA_2_DS_2_-VASc≥2)^12^ for stroke. About half (49.3%) of included patients were taking aspirin at the time of presentation.

### Main Results

Among the 138 OAC-naive patient-visits, 14.5% (n = 20) received a new prescription of warfarin or NOAC at discharge for stroke prophylaxis (Table 1). Other medications were not included in the analyses, but usage is detailed in Appendix Table A1.

### Provision of an Oral Anticoagulant Prescription Stratified By OAC-Naive Patients’ CHA_2_DS_2_-VASc and HAS-BLED Scores

OAC prescriptions were provided for 10.9% (n = 6) of patients with low stroke risk (CHA_2_DS_2_-VASc = 0 in males and 1 in females); 9.1% (n = 2) of patients with intermediate stroke risk (CHA_2_DS_2_-VASc = 1 in males); and 19.7% (n = 12) of patients with high stroke risk (CHA_2_DS_2_-VASc≥2) (Table 2).

**Figure 2:**
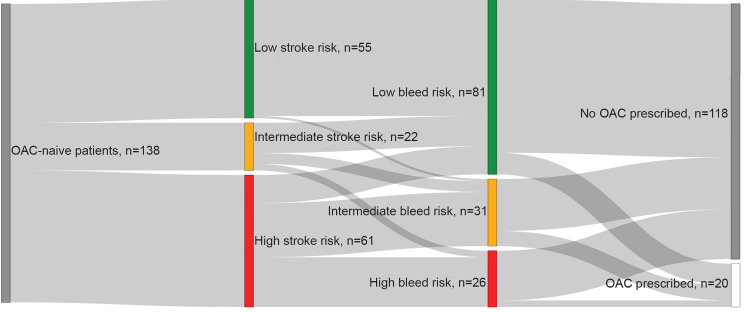
Patients who met exclusion criteria were stratified into low, intermediate, and high stroke risk by CHA_2_DS_2_-VASc score. They were then further stratified into low, intermediate, and high bleed risk by HAS-BLED scores. Next, they were stratified by prescription of oral anticoagulant (OAC) or not.

When stratified by HAS-BLED scores, OAC prescriptions were provided for 12.4% (n = 10/81) of patients with low bleeding risk, 22.6% (n = 7/31) of patients with intermediate bleeding risk, and 11.5% (n = 3/26) of patients with high bleeding risk. When stroke risk and bleeding risk were considered together, we found that patients with a high stroke risk and low bleeding risk (n = 13) were prescribed an OAC 15.4% (n = 2) of the time (Figure 2).

Among all those prescribed an OAC (any risk) (n = 20), 10.0% (n = 2) were at intermediate risk and 60.0% (n = 12) were at high risk for stroke. Among those at low risk of stroke (n = 55), 36.3% (n = 20) received aspirin and 10.9% (n = 6) received OACs. Of these low-risk patients prescribed aspirin, 95.0% (n = 19) were in normal sinus rhythm when they were discharged from the ED. Compared to the intermediate and high stroke risk patients who received an OAC prescription, we found that the low stroke risk patients prescribed an OAC were more likely to be younger (49.6 years vs 58.7 years), to be female (83% vs 57% male), to have private or commercial insurance (67% vs 43%), to present with a higher heart rate on arrival (137 vs 112), and have a shorter duration of symptoms, to have multiple methods of control attempted, to have cardioversion attempted (50% vs 7%), and were less likely to be on aspirin at the time of presentation (66.7% vs 33.3%).

### Predictors of OAC Prescription

Multivariable logistic regression showed that cardiology consultation and female gender were significant predictors of prescribing (Table 3). Females had 2.9 (95% confidence interval [CI], 1.0–8.5) times the odds of receiving an OAC prescription as compared to males, and patients with a cardiology consult had 12.5 (95% CI, 1.5–100.5) times the odds of receving an OAC prescription as compared to patients without a cardiology consult.

### Predictors of Cardiology Consultation

Cardiology was consulted in 64.5% of all cases. We identified hypertension as a significant predictor of cardiology consultation after controlling for duration of symptoms, insurance status, and comorbidities associated with CHA_2_DS_2_-VASc score calculation (Appendix Table A2). Patients with a diagnosis of hypertension had 2.7 (95% CI, 1.0–7.2) times the odds of having a cardiology consult compared with patients without hypertension.

### Cardiologists’ Recommendations for Oral Anticoagulant Prescription

For the 89 patients who received a cardiology consultation, we examined whether cardiology’s recommendation regarding OAC provision agreed with whether the ED provider prescribed an OAC. Cardiology recommended an OAC prescription for 10 (11.2%) patients, recommended against an OAC prescription for 40 (45.0%) patients, or opted to discuss OAC management at a later time for 19 (21.3%) patients (Appendix Table A3). Their recommendation was recorded as “unknown” for 20 (22.5%) patients. Other recommendations made by cardiology regarding patient management are specified in the appendix (Appendix Table A3).

### Agreement Between Cardiologists’ Recommendation for OAC And ED Provider Prescribing Patterns

For the 89 patients who received a cardiology consultation (36 of whom [40.5%] were high stroke risk), there were 12 cases in which cardiology’s recommendation was not congruent with the emergency physician’s decision (Appendix Table A4).

Cardiology recommended an OAC prescription for 10 of the 89 patients (11.2%), of whom seven were not prescribed an OAC. Cardiology did not recommend an OAC be prescribed to 40 patients, although five (12.5%) of these patients were prescribed an OAC by the emergency physician. We attempted to identify reasons for these discrepancies within the patients’ charts and identified one instance in which the ED provider opted against the recommended OAC prescription due to the patient’s low stroke risk, and another in which the ED provider prescribed an OAC after citing the patient’s high CHADS_2_ score (Appendix Table A1). Interestingly, patients who did not receive an OAC prescription despite cardiology’s recommendations were more likely to have a high HAS-BLED score (2/7 patients vs 0/5 patients who received an OAC prescription despite cardiology’s recommendation).

### Guidelines Cited by Provider

Of the 138 patient visits included, ED providers cited use of a clinical guideline such as CHA_2_DS_2_-VASc or HAS-BLED in AF management in 20.3% (n = 28) of visits. Use of a guideline was cited in 20.0% (n = 4) of visits where the patient was given an OAC prescription, and in 31.2% (n = 24) of visits where the patient was not prescribed an OAC or antiplatelet. Of all guidelines cited, CHADS_2_ was the most cited guideline, both for or against an OAC prescription. All patient visits were reviewed for evidence of reasons for/against OAC prescription other than use of a guideline.

### Identified Reasons for not Prescribing Oral Anticoagulant

We identified one visit in which the provider referenced the patient’s inability to follow up as an outpatient as a reason to support OAC prescription in the ED. Reasons against OAC prescription included low stroke risk (n=17), advanced age (n=4), lack of primary care physician management and/or follow-up (n=4), and “other” reasons (n=21). In patients perceived to be low stroke risk by the provider, 64.7% (11/17) were classified as low stroke risk by CHA_2_DS_2_-VASc. The most common “other” reason cited was that the patient was already taking aspirin (n=7).

### Oral Anticoagulant Prescribing Patterns

To evaluate changes in OAC prescribing patterns over time, we compared the types of OACs prescribed stratified by year in which the ED visit occurred (Appendix Table A5). There was no variation in warfarin vs NOAC prescriptions provided throughout the study period.

## DISCUSSION

In this study, we found that less than a quarter (15.3%) of OAC-naive AF patients at high risk for stroke and low risk for bleeding received a new prescription of warfarin or NOAC for stroke prophylaxis at the time of ED home discharge. This is consistent with findings from a previous study.^26^ Reasons for underutilization of OACs by emergency physicians for AF management are likely multifactorial.^27^–^30^ A recent qualitative study by our group found that physicians were uncomfortable with prescribing and had a sense of futility in prescribing due to concerns that included low adherence rates by patients prescribed anticoagulation and bleeding risks associated with anticoagulation,^31^ which are further magnified by an emergency physician’s inability to follow up with patients.

A longitudinal cohort study of United States and Canadian patients with new-onset AF found that use of warfarin decreased from 65% at study enrollment to 44% 30 months later.^29^ However, Atzema et al demonstrated that patients who received a prescription for warfarin in the ED had a higher frequency of long-term warfarin use than patients who were referred to another provider for OAC management.^32^ This suggests that there is longitudinal value in the initiation of a prescription associated with a significant event—an acute care encounter—and that more resources should be directed toward the initial acquisition of the medication for the patient. One potential solution by Barrett et al is the “provision of a protective tail of stroke prevention for a limited duration until they can follow up.”^33^

Interestingly, 10.9% (n = 6/55) of patients were over-prescribed OACs when they had a low stroke risk. This may be driven in part by the increased frequency of cardioversion attempted in this group (50% vs 7%), as anticoagulation is often continued for four weeks after electrical cardioversion and recommended by the American Heart Assocation.^15^ We also found that these patients were more likely to be younger, female, and have private or commercial insurance. However, these findings contradict those from a study of the Practice Innovation and Clinical Excellence (PINNACLE) Registry, which found that older age, male gender, and Medicare insurance were associated with increased likelihood of OAC prescription among AF patients with a CHA_2_DS_2_-VASc score of 0.^34^ The reason for this discrepancy is unclear, although our small sample size of six patients limits our ability to draw a statistically meaningful conclusion.

We found that cardiology consultation was a predictor of whether or not OAC-naive patients were prescribed an OAC on home discharge. These findings are in accordance with recently-published data from the non-oral vitamin K inhibitor era.^35^ Similarly, the TREAT-AF study found significant, specialty-dependent differences in anticoagulation use, with cardiologists being more likely to prescribe OACs than primary care physicians.^36^ This is likely due to provider comfort and familiarity with OAC prescribing. Additionally, having a cardiology consult may overcome barriers to outpatient follow-up as it directly connects the patient with a follow-up provider. Concern regarding lack of follow-up has been previously identified as a barrier to OAC prescription in the ED,^31^ and a lack of follow-up after ED discharge has been associated with increased mortality in AF patients.^26^,^30^,^37^

However, we also found that ED providers did not always abide by cardiology’s recommendations regarding OAC management, as management in the ED was incongruent with cardiology’s recommendations for 12 of 89 (13.5%) patients who received a cardiology consult. Although ED providers did not provide reasons for these discrepancies, patients who did not receive an OAC prescription, despite cardiology’s recommendations, were more likely to have a high-risk HAS-BLED score. While our simplified outcome maximized benefit and minimized harm (high stroke risk and low bleeding risk), we must acknowledge that a high bleeding-risk score does not preclude patients from being on OACs, and in fact, may still be indicated as the two risk scores share many features.

It is important to note that cardiology consults occurred in roughly two-thirds of encounters in our study population. This is higher than cardiology consults obtained in non-academic settings, with a recent study of Northern California Kaiser Permanente AF patients showing that cardiology was consulted 37.5% of the time.^35^ This reinforces the importance of improving emergency physician comfort with OAC prescribing independently of cardiology consultation.

Despite the fact that CHA_2_DS_2_-VASc and HAS-BLED risk scores are well-validated tools in the AF population, we found that they did not influence OAC prescribing. This reflects findings from a previous study that found only a modest correlation between CHADS_2_ score and warfarin prescribing in an elderly AF population.^38^ This may be because emergency physicians underutilize the tools (potentially due to unawareness of the guidelines), or because they overvalue the risk of adverse events (eg, major bleeding events) when considering OAC initiation. However, a recent multicenter prospective cohort study in Spain showed that anticoagulation initiated in the ED for AF patients with high stroke risk was not associated with an increase in major bleeding event by one year and was in fact associated with a decrease in mortality.^39^

We reviewed the reasons documented by physicians either for or against OAC prescription and found that use of a guideline was cited in only 20.3% of visits. This finding may suggest physicians’ unfamiliarity with risk-stratification tools not specifically intended for ED populations. A recent study reflected similar results, finding that among 1200 patients hospitalized at a community teaching institution with documented AF, only 14% had a CHA_2_DS_2_-VASc score documented in their charts.^13^,^40^ Those with a documented score were significantly more likely to have appropriate anticoagulation therapy, regardless of rate or rhythm control.^13^,^40^ Expanded efforts to educate emergency physicians on the use of these clinical decision-making tools may improve comfort with prescribing OACs, and thus improve time to appropriate anticoagulation.

This study contributes to the literature base describing NOAC-era ED prescribing practices for AF in OAC-naive patients.^26^,^35^ ED studies were limited to the use of warfarin until recently, but also show inappropriately low rates of OAC provision at ED discharge, ranging from less than one-quarter to nearly one-half of patients deemed eligible by calculation of stroke and bleeding risks.^26^,^32^,^35^,^41^ The number of patients prescribed NOACs is rapidly increasing, and it is critical to understand how this can inform clinical recommendations specific to the ED setting.^24^,^42^ Because our study took place over two years, we were able to evaluate changes in the rate of NOAC prescriptions over time and did not observe a significant change (Appendix Table A5). This is supported by a recent study showing the use of NOACs gradually increased over a three-year span (2012–2014); however, the use of warfarin was still 10–50 times more common than dabigatran, rivaroxaban, and apixaban as of 2015.^43^ In part, this may be due to challenges of prescribing NOACs from the ED as they often require prior authorization from a patient’s insurance.

Our work has again demonstrated an ED prescribing practice gap for anticoagulants in patients with a primary diagnosis of AF.^26^,^32^,^35^,^41^ However, it also showed that ED providers initiate OAC prescribing that may be incongruent with a cardiology consultation. Of note, while cardiology consultations influenced prescribing, they did not always correlate with the ED provider’s decision at the time of discharge. The inconsistencies in OAC prescribing are likely in part due to the lack of consensus guidelines for acute, ED-specific AF management, and has been previously noted in a qualitative study interviewing providers who were concerned about the lack of ED-specific guidelines as current guidelines use data from outpatient, chronic care populations.^14^,^31^,^44^ With no formal ED recommendations in place, it is not surprising that more than half of patients with AF and high stroke risk do not receive an OAC prescription at the time of home discharge.^41^

A lack of guideline utilization by providers may include (1) wariness of using scoring tools that are not specifically validated in ED populations; (2) hesitancy to start aggressive anticoagulation therapy without definitive follow-up; (3) over-reliance on cardiology consultants; and (4) lack of education regarding clinical decision-making tools (CHA_2_DS_2_-VASc and HAS-BLED), as well as other reasons.^31^ There is an opportunity to engage emergency physicians to validate existing clinical algorithms for AF management in ED populations. Systems-specific interventions and electronic clinical decision support could include improved methods for establishing outpatient follow-up after ED evaluation. These are several of many ways emergency clinicians can be empowered to contribute to multidisciplinary efforts to prevent strokes in patients with high-risk AF.^45^

## LIMITATIONS

Patients were included only if they had a primary ED diagnosis of AF, and therefore the conclusions from this study may not be applicable to patients with a different primary diagnosis accompanied by AF (e.g., a patient with pneumonia noted to have incidental AF). Patients with related diagnoses such as atrial flutter were not included. The degree of valvular disease was not abstracted. In addition, we included only patients who were discharged home from the ED. As a result, our patient population may have reflected patients with lower stroke and/or bleeding risk (determined by CHADS_2_, CHA_2_DS_2_-VASc, and HAS-BLED tools), fewer co-morbidities, and a more favorable disposition status.

This retrospective study is limited to one academic, tertiary care, urban hospital and our results may be influenced by regional and/or institution-specific practice patterns, and our analysis is limited by what was available in the EHR. Prospective validation and external validation at other EDs is needed.

## CONCLUSION

Our study suggests that current risk stratification tools for AF management are ineffectively used in the ED. Cardiology consultation and female sex were the only variables associated with OAC prescription at discharge. This may be explained by ED providers’ unfamiliarity with risk-stratification tools, lack of comfort with OAC prescribing, or inability to facilitate patient follow-up. Clear guidelines for ED providers are critical in this high-risk and undertreated population. Possible solutions include new algorithms, expanded educational dissemination of existing guidelines, or collaborating with cardiology departments to create protocols for initiation of anticoagulation by ED providers coupled with automatic and timely outpatient follow-up for longitudinal management.

## Supplementary Information



## Figures and Tables

**Table 1:** Patient characteristics and calculated stroke and bleeding risk scores for 138 OAC-naive atrial fibrillation patients who were discharged home from the ED.

Characteristic (n, %)	Overall (n=138,100%)	OAC Prescription (n=20,14.5%)	No OAC (n=118,85.5%)	p-value*
Age (years), mean (SD)	58.7 (17.1)	61.4 (13.8)	58.2 (17.6)	0.69
Female gender	54 (39.1%)	13 (65.0%)	41 (34.7%)	0.01
Race				
White	128 (92.8%)	20 (100.0%)	108 (91.5%)	1.00
Black or African American	3 (2.2%)	0 (0.0%)	3 (2.5%)	
Asian or Pacific Islander	2 (1.4%)	0 (0.0%)	2 (1.7%)	
Other	2 (1.4%)	0 (0.0%)	2 (1.7%)	
Not reported	3 (2.2%)	0 (0.0%)	3 (2.5%)	
Insurance				
Commercial	59 (42.8%)	10 (50.0%)	49 (41.5%)	0.24
Medicare/Medicaid	64 (46.4%)	10 (50.0%)	54 (45.8%)	
Other	15 (10.9%)	0 (0.0%)	15 (12.7%)	
History of AF	81 (58.7%)	10 (50.0%)	71 (60.2%)	0.29
Symptom onset				
< 6 hours	64 (46.4%)	11 (55.0%)	53 (44.9%)	0.05
6–48 hours	28 (20.3%)	3 (15.0%)	25 (21.2%)	
> 48 hours	10 (7.2%)	4 (20.0%)	6 (5.1%)	
Unknown	36 (26.1%)	2 (10.0%)	34 (28.8%)	
Heart rate on arrival, mean (SD)	118 (31.5)	112 (30.3)	119 (31.7)	
Rate-controlling medication PTA	63 (45.7%)	12 (60.0%)	51 (43.2%)	0.16
On aspirin prior to presentation	68 (49.3%)	12 (60.0%)	56 (47.5%)	0.30
CHA_2_DS_2_-VASc group†				
Low stroke risk	55 (39.9%)	6 (30.0%)	49 (41.5%)	0.30
Intermediate stroke risk	22 (15.9%)	2 (10.0%)	20 (16.9%)	
High stroke risk	61 (44.2%)	12 (60.0%)	49 (41.5%)	
HAS-BLED group§				
Low bleeding risk	81 (58.7%)	10 (50.0%)	71 (60.2%)	0.42
Intermediate bleeding risk	31 (22.5%)	7 (35.0%)	24 (20.3%)	
High bleeding risk	26 (18.8%)	3 (15.0%)	23 (19.5%)	
Number of methods of control attempted				
0	57 (41.3%)	10 (50.0%)	47 (39.8%)	0.19
1	52 (37.7%)	4 (20.0%)	48 (40.7%)	
2	21 (15.2%)	4 (20.0%)	17 (14.4%)	
3	6 (4.3%)	1 (5.0%)	5 (4.2%)	
4	2 (1.4%)	1 (5.0%)	1 (0.8%)	
First method of control				
Rhythm	16 (11.6%)	1 (5.0%)	15 (12.7%)	0.52
Rate	65 (47.1%)	9 (45.0%)	56 (47.5%)	
None	57 (41.3%)	10 (50.0%)	47 (39.8%)	
Cardioversion attempted	18 (13.0%)	4 (20.0%)	14 (11.9%)	0.30

* t-tests for continuous data, chi-square tests for categorical data, and Fisher’s exact tests for sparse categorical data.

† CHA_2_DS_2_-VASc (congestive heart failure, hypertension, age≥75, diabetes mellitus, prior stroke or transient ischemic attack, gender, age 65–74 years, and vascular disease). 0 in males, 1 in females = low risk for stroke, 1 in males = intermediate risk, and ≥ 2 high risk.

§ HAS-BLED (hypertension, abnormal renal function or liver function, stroke, bleeding, labile international normalized ratio [excluded as all patients not on warfarin prior to inclusion], elderly >85 years old, and drugs and alcohol): 0 = low risk, 1 to 2 = moderate risk, >2 = high risk.

*OAC*, oral anticoagulant; *AF*, atrial fibrillation; *SD*, standard deviation; *PTA*, prior to arrival.

**Table 2:** Provision of OAC prescription by CHA_2_DS_2_-VASc and HAS-BLED score.

CHA_2_DS_2_-VASc score†	HAS-BLED score§	OAC Prescription		

		Yes (n=20)	No (n=118)	Total (n=138)
				
Low stroke risk				
	Low bleeding risk	6 (11.1%)	48 (88.9%)	54 (100%)
	Intermediate bleeding risk	0 (0%)	1 (100%)	1 (100%)
	High bleeding risk	0 (0%)	0 (0%)	0 (0%)
	Total	6	49	55
Intermediate stroke risk				
	Low bleeding risk	2 (14.3%)	12 (85.7%)	14 (100%)
	Intermediate bleeding risk	0 (0%)	5 (100%)	5 (100%)
	High bleeding risk	0 (0%)	3 (100%)	3 (100%)
	Total	2	20	22
High stroke risk				
	Low bleeding risk	2 (15.4%)	11 (84.6%)	13 (100%)
	Intermediate bleeding risk	7 (28.0%)	18 (72.0%)	25 (100%)
	High bleeding risk	3 (13.0%)	20 (87.0%)	23 (100%)
	Total	12	49	61

† CHA_2_DS_2_-VASc (congestive heart failure, hypertension, age ≥ 75, diabetes mellitus, prior stroke or transient ischemic attack, gender, age 65–74 years, and vascular disease). 0 in males, 1 in females = low risk for stroke, 1 in males = intermediate risk, and ≥ 2 high risk.

§ HAS-BLED (hypertension, abnormal renal function or liver function, stroke, bleeding, labile international normalized ratio [excluded as all patients not on warfarin prior to inclusion], elderly >85 years old, and drugs and alcohol): 0 = low risk, 1 to 2 = moderate risk, >2 = high risk.

*OAC*, oral anticoagulant; *AF*, atrial fibrillation.

**Table 3:** Factors associated with the provision of oral anticoagulant prescription at ED home discharge to 67 (48.2%) of 138 OAC-naive AF patients.

Characteristic	OR (95% CI)	P-value
Gender, Female	2.9 (1.0–8.5)	**0.05**
CHA_2_DS_2_-VASc stratification		
High risk	1.9 (0.7–5.7)	0.21
Low/intermediate risk	referent	
Cardiology consultation	12.5 (1.5–100.5)	**< 0.01**

Significant values are bolded.

*OR*, odds ratio; *CI*, confidence interval; *AF*, atrial fibrillation.
