# Supplementary material for: Practice Gap in Atrial Fibrillation Oral Anticoagulation Prescribing at Emergency Department Home Discharge
Source: West J Emerg Med. 2020 Jun 29;21(4):924–34. doi: 10.5811/westjem.2020.3.45135 (PMC7390546; doi:10.5811/westjem.2020.3.45135)
Supplement: Supplementary file 1 [file wjem-21-924-s001.docx]

**Appendix Tables A1 – A5**

Kea B, et al. Practice Gap in Atrial Fibrillation Oral Anticoagulation Prescribing at Emergency Department Home Discharge. *West J Emerg Med*. 2020. Doi: 10.5811/westjem.2020.3.45135

**Table A1.** Patients with a discrepancy between cardiology’s recommendation and ED provider prescribing.

| **Patient** | **OAC Rx recommended by cardiology** | **OAC Rx provided by ED provider** | **CHA_2_DS_2_-VASc** | **HASBLED** | **Rx given** | **Reason for discrepancy?** |
| --- | --- | --- | --- | --- | --- | --- |
| 1 | Yes | No | High Risk | High Risk | N/A | N/A |
| 2 | Yes | No | Low Risk | Low Risk | N/A | “Low stroke risk” |
| 3 | Yes | No | High Risk | Low Risk | N/A | N/A |
| 4 | Yes | No | High Risk | High Risk | N/A | N/A |
| 5 | Yes | No | Low Risk | Low Risk | N/A | N/A |
| 6 | Yes | No | High Risk | Intermediate | N/A | N/A |
| 7 | Yes | No | Low Risk | Low Risk | N/A | N/A |
| 8 | No | Yes | Intermediate | Low Risk | Warfarin | “CHADS_2_ score” |
| 9 | No | Yes | Low Risk | Low Risk | Warfarin | N/A |
| 10 | No | Yes | High Risk | Intermediate | Dabigatran | N/A |
| 11 | No | Yes | High Risk | Intermediate | Other | N/A |
| 12 | No | Yes | Low Risk | Low Risk | Dabigatran | N/A |

*OAC,* Oral anticoagulation; *Rx*, Prescription; *ED,* Emergency Department.

**Table A2.** Predictors of a cardiology consult in 89 OAC-naïve AF patients.

| **Characteristic** | **OR (95% CI)** | **P- Value** |
| --- | --- | --- |
| **Sex, Female** | 1.6 (0.6-4.2) | 0.30 |
| **Duration of AF** |  | 0.45 |
| 6-48 hours | 2.2 (0.7-7.3) |  |
| >48 hours | 0.6 (0.1-3.0) |  |
| Unknown | 1.0 (0.4-2.9) |  |
| <6 hours | referent |  |
| **Insurance** |  |  |
| Medicare/Medicaid | 0.4 (0.1-1.3) | 0.25 |
| Other | 0.4 (0.1-1.6) |  |
| Commercial | referent |  |
| **Congestive Heart Failure** | 0.4(0.1-1.6) | 0.19 |
| **Hypertension** | 2.7(1.0-7.2) | 0.04 |
| **Age** |  |  |
| <65 | 0.5(0.1-1.7) | 0.49 |
| 65-74 | 0.6(0.2-2.4) |  |
| ≥75 | referent |  |
| **Diabetes** | 0.3 (0.1–1.1) | 0.06 |
| **Previous Stroke** | 0.5(0.1-2.1) | 0.33 |
| **Vascular Disease** | 2.8(0.4-18.0) | 0.28 |

*OAC*, oral anticoagulant; *AF*, atrial fibrillation; *OR*, odds ratio; *CI,* confidence interval.

**Table A3.** OAC prescription recommendation by cardiology.

| **OAC recommendation** | **n (%)** |
| --- | --- |
| **Yes** | 10 (11.2) |
| **No** | 40 (45.0) |
| **To be discussed later** | 19 (21.3) |
| **Unknown** | 20 (22.5) |
| **Total** | 89 (100) |
| **Other reasons noted in patients’ charts** | 17 |
| ASA 325mg daily | 4 |
| Follow up with outpatient cardiology | 2 |
| Daily ASA & beta blocker recommended | 2 |
| ASA 81mg daily | 1 |
| Flecainide vs beta blocker recommended | 1 |
| Patient spontaneously converted to NSR | 1 |
| Ablation to be discussed later | 1 |
| “Doesn’t need it since patient doing well” | 1 |
| Increase dose of current beta blocker | 1 |
| AF in the setting of heavy alcohol consumption | 1 |
| Low *CHA_2_DS_2_-VASc* score | 1 |
| Single dose Flecainide recommended | 1 |

*OAC,* oral anticoagulant; *ASA,* Aspirin; *AF,* Atrial fibrillation.

**Table A4.** Cardiology OAC recommendation vs ED provider OAC prescribing.

| **OAC prescription recommended by cardiology?** | **OAC prescription provided by ED provider?** | | |
| --- | --- | --- | --- |
|  | **Yes n (%)** | **No n (%)** | **Total** |
| Yes | 3 (30.0) | 7 (70.0) | 10 |
| No | 5 (12.5) | 35 (87.5) | 40 |
| To be discussed later | 5 (26.3) | 14 (73.7) | 19 |
| Unknown | 5 (25.0) | 15 (75.0) | 20 |
| Total | 18 | 71 | 89 |

*OAC,* Oral anticoagulation; *ED,* Emergency Department.

**Table A5.** Anticoagulation and antiplatelet prescription practices over time.

| **Medication prescribed** | **2012**  **n** | **2013**  **n** | **2014**  **n** | **Total**  **n** |
| --- | --- | --- | --- | --- |
| Apixaban | 0 | 0 | 1 | 1 |
| Warfarin +/- enoxaparin bridge | 6 | 3 | 2 | 11 |
| Enoxaparin | 1 | 0 | 0 | 1 |
| Dabigatran | 3 | 3 | 3 | 9 |
| Clopidogrel | 0 | 0 | 1 | 1 |
| Aspirin | 18 | 12 | 18 | 48 |
| Other: |  |  |  |  |
| Aspirin + either dabigatran or warfarin  depending on insurance coverage | 1 | 0 | 0 | 1 |

“Other” to include regimens not fitting into clearly defined anticoagulants or antiplatelet strategies.
